# Comparative Immunogenicity of the Tetanus Toxoid and Recombinant Tetanus Vaccines in Mice, Rats, and Cynomolgus Monkeys

**DOI:** 10.3390/toxins8070194

**Published:** 2016-06-25

**Authors:** Rui Yu, Ting Fang, Shuling Liu, Xiaohong Song, Changming Yu, Jianmin Li, Ling Fu, Lihua Hou, Junjie Xu, Wei Chen

**Affiliations:** Laboratory of Vaccine and Antibody Engineering, Beijing Institute of Biotechnology, 20 Dongdajie Street, Fengtai District, Beijing 100071, China; yurui1102@139.com (R.Y.); fangting1008vip@163.com (T.F.); lslsjy2203@sina.com (S.L.); songxiaohong78@sina.com (X.S.); yuchangming@126.com (C.Y.); jianminli@126.com (J.L.); fuling@139.com (L.F.)

**Keywords:** tetanus toxin, recombinant vaccine, toxoid vaccine, immunogenicity, booster vaccination efficacy

## Abstract

Tetanus is caused by the tetanus neurotoxin (TeNT) and is one of the most dreaded diseases especially in the developing countries. The current vaccine against tetanus is based on an inactivated tetanus toxin, which is effective but has many drawbacks. In our previous study, we developed a recombinant tetanus vaccine based on protein TeNT-Hc, with clear advantages over the toxoid vaccine in terms of production, characterization, and homogeneity. In this study, the titers, growth extinction, and persistence of specific antibodies induced by the two types of vaccine in mice, rats, and cynomolgus monkeys were compared. The booster vaccination efficacy of the two types of vaccines at different time points and protection mechanism in animals were also compared. The recombinant tetanus vaccine induced persistent and better antibody titers and strengthened the immunity compared with the commercially available toxoid vaccine in animals. Our results provide a theoretical basis for the development of a safe and effective recombinant tetanus vaccine to enhance the immunity of adolescents and adults as a substitute for the current toxoid vaccine.

## 1. Introduction

Tetanus is characterized by the neurotoxicity of the anaerobic bacterium *Clostridium tetani* [1]. The neurotoxin has an estimated lethal dose of <2.5 ng/kg (WHO, 2010). The dramatic muscular spasms of tetanus make it one of the world’s most feared diseases, especially in the developing countries. The worldwide mortality rates of tetanus range from 6% to 72%, depending on the medical condition in developing countries [2]. In 2010, it caused about 61,000 deaths [3] and WHO estimates that 58,000 newborns died from neonatal tetanus (http://www.who.int/immunization_monitoring/diseases/MNTE_initiative/en/index.html). Natural calamities such as earthquakes, landslides, mud-rock flows, floods, and typhoons increase the risk of tetanus. Two and a half weeks after the Indian Ocean tsunami, there were 106 tetanus cases in Aceh, Indonesia, including 20 deaths. Tetanus cases were also reported after the earthquake in Pakistan in 2005.

The currently available tetanus vaccine is based on inactivated tetanus toxin and is extremely effective in protecting against tetanus [4]. However, the existing tetanus toxoid vaccine is associated with toxicity, side effects, production dangers, and pollution caused by formaldehyde. Effective and safe prophylactic vaccines against tetanus are under investigation in many countries.

The recombinant vaccine contains proteins genetically engineered to induce a protective immune response clinically or pre-clinically. Compared with the traditional inactivated vaccine, the recombinant version contains fewer or no pyrogens, without any immune suppression or harmful reaction. The recombinant protein is also easily produced and purified on a large scale with high-quality control and safety. The Hc domain of tetanus neurotoxin (TeNT-Hc), which is also called TTFc (Tetanus Toxin Fragment C) and retains the full binding affinity to neuronal cells via gangliosides in lipid rafts [5,6,7], shows considerable promise as a possible next-generation subunit vaccine against tetanus [8,9,10,11,12,13,14,15].

In our previous work, we successfully developed a recombinant tetanus vaccine based on protein TeNT-Hc. The non-tagged TeNT-Hc exhibited excellent soluble expression in *E. coli* with a final yield of about 333 mg/L in a 42-L pilot scale after three-step purification. To investigate the development of the vaccine for human use, aluminum hydroxide adjuvant was added to adsorb the protein TeNT-Hc. The excellent immunogenicity of the recombinant tetanus vaccine and protection in mice was also established [16]. Testing for acute toxicity in mice, reproductive developmental toxicity in rats, systemic active allergy in guinea pigs, and long-term toxicity in cynomolgus monkeys was conducted in a GLP-certified laboratory. The results showed that the recombinant tetanus vaccine was safe (data unpublished).

To further study the feasibility of the recombinant tetanus vaccine as a substitute for the currently available tetanus toxoid vaccine, we compared the titers and persistence of specific antibodies induced by the two types of vaccines in mice, rats, and cynomolgus monkeys. We also compared the potency of the two kinds of vaccines as a booster at different time points. The protective effects of the two vaccines in animals were also compared. Our results provided a experimental basis for the safety and efficacy of the recombinant tetanus vaccine for the development of the next-generation vaccine against tetanus.

## 2. Results

### 2.1. Long-Term Immunogenicity of the Recombinant Tetanus Vaccine and the Tetanus Toxoid Vaccine

#### 2.1.1. Long-Term Serological Comparison in Mice

**Figure 1:**
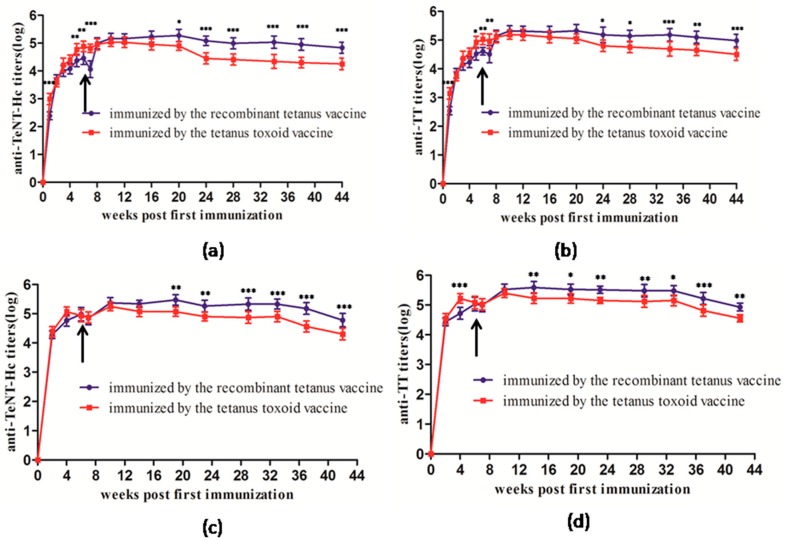
Kinetics of anti-TeNT-Hc and anti-TT antibody titer development in mice (**a**,**b**) and rats (**c**,**d**) following immunization with the two different vaccines. Each BALB/c mouse (6 per group) or Wistar rat (6 per group) was immunized with 0.5 mL of different vaccines at Week 0 and again when the antibody titer decreased (at Week 7). All the mice were bled at different time points until 44 weeks and all the rats were bled until 42 weeks for the ELISA with TeNT-Hc or TT as coated protein. Each point represents the mean reciprocal log 10 endpoint titer and standard error. A Student’s *t* test was used to determine the significance of the difference between two independent data, and two-way ANOVA was used to determine the significance between the two groups. (* 0.01 < *p* < 0.05; ** 0.001 < *p* < 0.01; *** *p* < 0.001).

BALB/c mice were immunized twice with the recombinant tetanus vaccine and the tetanus toxoid vaccine, respectively. After the first immunization, the antibody titers were detected every week and the second immunization was carried out when the antibody titers were decreased. As shown in Figure 1a,b, the increase and decline in antibody levels by the two kinds of vaccines in mice were basically similar. The anti-TeNT-Hc and anti-TT titers peaked at Week 6 and were elevated further after the second immunization at Week 7. Both groups of mice maintained a high antibody titer until Week 44. The anti-TeNT-Hc and anti-TT antibody titers in the toxoid vaccine group were higher than in the recombinant vaccine group from Weeks 5 to 7. However, the antibody levels in the mice of the recombinant vaccine group showed a rapid rise after the second immunization and were significantly higher than in the toxoid vaccine group with the anti-TeNT-Hc antibodies from the beginning of Week 20 and the anti-TT antibodies from Week 24. The above results suggested that the recombinant tetanus vaccine induced an antibody pattern similar to the toxoid vaccine in mice, but the recombinant vaccine was superior to the toxoid vaccine in terms of booster effect.

#### 2.1.2. Long-Term Serological Comparison in Rats

Wistar rats were immunized twice by the recombinant tetanus vaccine and the tetanus toxoid vaccine, respectively. After the first immunization, the antibody titers were checked every week and the second immunization was carried out when the antibody titers were decreased. As shown in Figure 1c,d, the anti-TeNT-Hc and anti-TT titers of the tetanus toxoid vaccine group peaked at Week 4 and the recombinant tetanus vaccine group peaked at Week 6. The antibody titers of both groups were elevated after the second immunization at Week 7 and were maintained at a high level until Week 42. The anti-TeNT-Hc antibody titers of the recombinant tetanus vaccine group, which showed no significant difference from the toxoid group from Weeks 0 to 14, were higher than those of the toxoid vaccine group from Weeks 19 to 42. The anti-TT antibody titers of the recombinant tetanus vaccine group were significantly higher than that of the toxoid vaccine group from Weeks 14 to 42, while they were lower at Week 4 and showed no significant difference from the toxoid group from Weeks 6 to 10. The above results suggested that the recombinant tetanus vaccine induced persistent antibody titers and strengthened immunization better than the commercially available toxoid vaccine in rats.

### 2.2. Comparative Immunogenicity in Cynomolgus Monkeys

**Figure 2:**
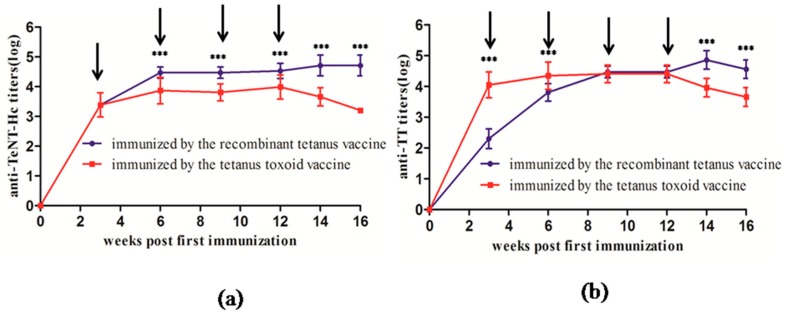
Kinetics of anti-TeNT-Hc (**a**) and anti-TT (**b**) antibody titer development in cynomolgus monkeys following immunization with two different vaccines. Each monkey (10 per group) was immunized with 0.5 mL of different vaccines at Weeks 0, 3, 6, 9, and 12 and was bled at Weeks 0, 3, 6, 9, 12, 14, and 16 for the ELISA with TeNT-Hc or TT as the coated protein. Each point represents the mean reciprocal log 10 endpoint titer and standard error. A Student’s *t* test was used to determine the significance of the difference between two independent data, and two-way ANOVA was used to determine the significance between the two groups. *** *p* < 0.001.

Multiple immunization using recombinant tetanus vaccine and tetanus toxoid vaccine was compared in cynomolgus monkeys. Each monkey received vaccination five times at three-week intervals. As shown in Figure 2, the anti-TeNT-Hc antibody titers of the recombinant tetanus vaccine group were significantly higher than those of the tetanus toxoid vaccine group since the second vaccination. The anti-TT antibody titers of the recombinant tetanus vaccine group were lower than those of the tetanus toxoid vaccine group from Weeks 3 to 6, but there was no significant difference between the two groups from Weeks 9 to 12. The anti-TT antibody titers of the recombinant tetanus vaccine group were significantly higher than those of the tetanus toxoid vaccine group from two weeks after the last immunization. After the last vaccination, specific antibody titers showed significant decline in the toxoid vaccine group, while the recombinant vaccine group still maintained a high antibody titer. The above results showed that the recombinant tetanus vaccine had excellent performance in strengthening the immunogenicity and persistence compared with the toxoid vaccine.

### 2.3. Comparision of the Booster Immunization between Recombinant Tetanus Vaccine and Tetanus Toxoid Vaccine

The results of long-term immunogenicity indicated that the recombinant vaccine had a better immune-strengthening effect in animals. Considering that most people receive basic immunization for diphtheria pertussis tetanus (DPT) vaccine and tetanus vaccine is mainly used to strengthen the immunity in adolescents and adults, we designed two experiments to further compare the booster effect of the two types of vaccines, respectively.

**Figure 3:**
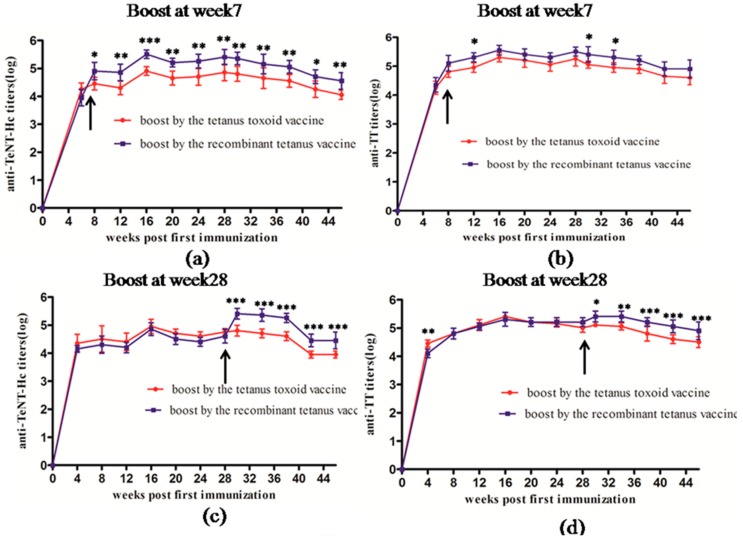
Kinetics of anti-TeNT-Hc and anti-TT antibody titer development in mice following booster immunization with two different vaccines at Week 7 (**a**,**b**) or Week 28 (**c**,**d**). When boosting at Week 7, each mouse (6 per group) was immunized with 0.5 mL of the tetanus toxoid vaccine at Week 0 and a 0.5-mL booster dose of the recombinant tetanus vaccine and the tetanus toxoid vaccine at Week 7, respectively. When boosting at Week 28, each mouse was immunized with 0.5 mL of the tetanus toxoid vaccine at Weeks 0 and 7, and a 0.5-mL booster dose of recombinant tetanus vaccine and tetanus toxoid vaccine at Week 28, respectively. All mice were bled at different time points until 46 weeks for the ELISA with TeNT-Hc or TT as the coated protein. Each point represents the mean reciprocal log 10 endpoint titer and standard error. A Student’s *t* test was used to determine the significance of the difference between two independent data, and two-way ANOVA was used to determine the significance between the two groups. (* 0.01 < *p* < 0.05; ** 0.001 < *p* < 0.01; *** *p* < 0.001).

When boosting at Week 7 using different vaccines, as shown in Figure 3a,b, the recombinant tetanus vaccine showed excellent performance in anti-TeNT-Hc and anti-TT titers. The anti-TeNT-Hc antibody titers of the recombinant vaccine group were significantly higher than those of the toxoid vaccine group from Weeks 12 to 46. The anti-TT antibody titers of the recombinant vaccine group were also significantly higher than those of the toxoid vaccine group at Weeks 12, 30, and 34, while it was slightly higher at other time points after the booster immunization without a significant difference.

When boosting at Week 28 using different vaccines after two basic vaccinations with the tetanus toxoid at Weeks 0 and 7, as shown in Figure 3c,d, the anti-TeNT-Hc and anti-TT antibody titers of the recombinant vaccine group were both significantly higher than those of the toxoid vaccine group from Weeks 30 to 46. This result suggested an excellent immune enhancing effect of the recombinant tetanus vaccine.

### 2.4. Long-Term In Vivo Protection of Vaccinated Mice and Rats

The potencies of the recombinant tetanus vaccine and the tetanus toxoid vaccine in mice and rats, which were immunized twice at Weeks 0 and 7 were studied. All the mice including a group vaccinated by PBS were challenged with 2 × 10^3^ LD50s of tetanus neurotoxin 44 weeks after first immunization, while all the rats including a group vaccinated with PBS were challenged with 2 × 10^3^ LD50s of tetanus neurotoxin 42 weeks after first immunization. As shown in Table 1, the two types of vaccine provide 100% protection in mice and rats, while the animals in the negative control groups died.

### 2.5. Potency Comparison of Low-Dose Vaccines in Mice

**Figure 4:**
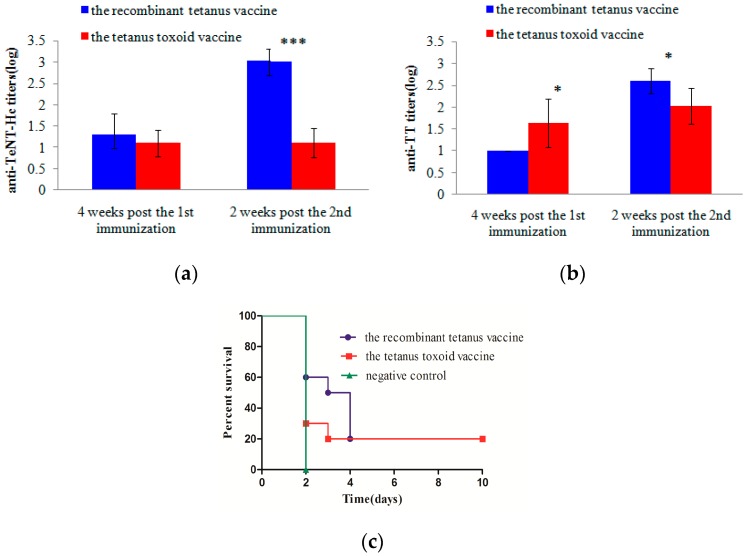
Efficacy comparison in mice following two different low-dose vaccinations. Each BALB/C mouse (10 per group) was immunized with 0.5 mL of 1000× diluted vaccines at Weeks 0 and 4 and was bled at Weeks 4 and 6 for the ELISA with TeNT-Hc (**a**) or TT (**b**) as the coated protein. Each point represents the mean reciprocal log 10 endpoint titer and standard error. A Student’s *t* test was used to assess the statistical significance of difference among the different vaccine groups. All the mice were challenged with 100 LD50s of tetanus neurotoxin two weeks after the second vaccination. The animals that died within 10 days after the challenge were recorded. One group of mice was immunized by PBS and the adjuvant as a negative control (**c**). Survival curves were compared using a log rank (Mantel–Cox) test. (* 0.01 < *p* < 0.05; *** *p* < 0.001).

The immunogenicity and protective effect of low-dose recombinant tetanus vaccine and the tetanus toxoid vaccine were compared in mice. Each group of mice was immunized twice with a 0.5-mL 1000-fold diluted vaccine at Weeks 0 and 4, respectively. The anti-TeNT-Hc and anti-TT antibody titers were detected and compared four weeks after the first immunization and two weeks after the second immunization. As shown in Figure 4a,b, four weeks after the first vaccination, the recombinant vaccine group was similar to the toxoid vaccine group in the anti-TeNT-Hc antibody titers (*p* > 0.05), but was lower than the toxoid vaccine group in the anti-TT antibody titers (*p* < 0.05). However, two weeks after the second vaccination, the recombinant vaccine group was much higher than the toxoid vaccine group both in the anti-TeNT-Hc titers (*p* < 0.001) and the anti-TT titers (*p* < 0.05). All mice were first challenged with 50 LD50s TeNT two weeks after the second vaccination, and the two groups all showed 100% protections. When challenged with 100 LD50s of TeNT, the death of the mice in the recombinant vaccine group was slightly delayed compared with those in the toxoid vaccine group (Figure 4c). However, the survival rate of the two groups was not significantly different (*p* > 0.05).

### 2.6. Neutralizing Antibodies in the Vaccinated Cynomolgus Monkeys

**Figure 5:**
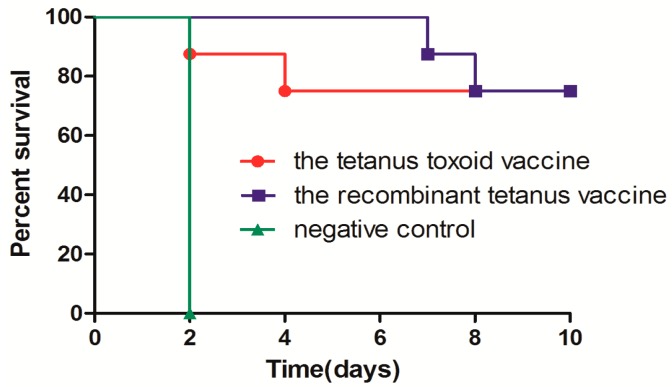
Neutralizing activities of the antibodies induced by the two vaccines in cynomolgus monkeys. A volume of 0.5 μL of serum of the cynomolgus monkeys, immunized four weeks after the fifth time, was mixed with 20 LD50s of tetanus neurotoxin and injected intraperitoneally into BALB/C mice (8 per group). All mice were monitored for 10 days, and those alive were scored as survivors. One group of mice, which was only challenged with 20 LD50s of tetanus neurotoxin, was used as the negative control. Survival curves were compared using a log rank (Mantel-Cox) test.

The serological neutralization in the vaccinated cynomolgus monkeys following administration of the recombinant tetanus vaccine and the tetanus toxoid vaccine was studied. A volume of 0.5 mL of serum from the cynomolgus monkeys, which were vaccinated four weeks after the fifth time, was mixed with 20 LD50s of tetanus neurotoxin. The mixture was injected into mice, and the survival rates were calculated. As shown in Figure 5, both the protective rates of serum from each kind of vaccine reached 75%. However, compared with the toxoid vaccine group, the serum from the recombinant vaccine group significantly delayed death in mice (*p* < 0.01).

## 3. Discussion

TeNT is an approximately 150 kDa protein consisting of a 50-kDa light chain, an active site for proteolysis, and a 100-kDa heavy chain. The heavy chain is composed of a translocation (H_N_) and a binding (Hc) domain, each of approximately 50 kDa [16,17,18]. TT was capable of inducing the production of antibodies not only against TeNT-Hc but also against H_N_ and light chain. However, the antibodies against TeNT-Hc induced by TT are the main neutralizing antibodies for protection. The TeNT-Hc contains the carboxy terminal half of the heavy chain [19]. The recombinant TeNT-Hc mediates both the specific binding and internalization into the spinal cord neuron cultures [20]. The TeNT-Hc is non-toxic and has been studied as a potential candidate for vaccine against tetanus. This type of vaccine has advantages over traditional toxin-inactivated vaccines in that it is cheaper, less hazardous to produce, and can be generated in large quantities. TeNT-Hc has been cloned and expressed by others; however, poor solubility resulted in low yields and difficulties associated with the purification of the expressed protein in *E. coli* limited the utility of TeNT-Hc. Many studies have been carried out to get high expression of TeNT-Hc, but they were not accepted by the industry because of poor efficacy compared with commercial toxoid vaccines. In our previous work [16], we developed a recombinant tetanus vaccine based on protein TeNT-Hc using our patented technology (Chinese invention patent No: ZL200910135972.0), which is the first report on the purification of a non-tag TeNT-Hc isoform from *E. coli* in large quantities for potential human application. The soluble expression of TeNT-Hc suggested a structural resemblance to the natural form compared with the insoluble inclusion bodies and therefore resulted in better immunogenicity. This type of recombinant vaccine also displayed advantages over traditional toxin-inactivated vaccines as a cost-effective and safer version that can be generated in large quantities.

The DPT vaccine containing the tetanus toxoid vaccine has been universally used in infants and children for many years to effectively prevent tetanus. However, a comparative study of the current toxoid vaccine and the recombinant tetanus vaccine was unavailable.

In this study, we compared the growth and decline in antibodies induced by the two types of vaccines in mice and rats. The results showed that the recombinant vaccine elicited similar antibody response to the toxoid vaccine. The anti-TeNT and anti-TT antibody titers peaked 4 to 6 weeks after the first immunization, and a second vaccination evoked a higher immune response. This result was also consistent with the immunization schedule of the toxoid vaccine, spanning 4 to 8 weeks between the first and the second vaccination.

Comparison of the long-term immunogenicity between the recombinant tetanus vaccine and the tetanus toxoid vaccine indicated that the recombinant vaccine showed better efficacy as a booster in mice, rats, and cynomolgus monkeys. Most people receive basic immunization with the DPT vaccine, and the single tetanus vaccine is mainly used to strengthen the immunity in adolescents and adults. Therefore, we designed two experiments to further compare the efficacy of booster vaccination using the two types of vaccines, severally. The administration of a booster at Week 7 as the second vaccination or booster at Week 28 for the third vaccination using the two types of vaccines showed that the recombinant tetanus vaccine had a better performance than the toxoid vaccine in antibody titers and persistence. Furthermore, the immunization program of the tetanus vaccine in humans is three instances of the basic immunization and a booster immunization every 5–10 years. In our study, we found that mice and rat could maintain a high titer antibody levels after two immunizations without significant titer decreasing. Thus, we thought it was not necessary to perform the third vaccination in mice and rats. Meanwhile, in the more expensive cynomolgus monkey model, five instances of immunization were carried out to investigate not only the immunogenicity but also the long-term toxicity and safety of the vaccine.

In vivo protection after long-term immunization showed protection by the two kinds of vaccines in mice or rats against 2 × 103 LD50s of tetanus neurotoxin. The protection of the low-dose recombinant tetanus vaccine and the tetanus toxoid vaccine were also compared in mice. The death of mice in the recombinant vaccine group was slightly delayed compared with those in the toxoid vaccine group. However, the survival rate of the two groups showed no significant difference. Compared with the toxoid vaccine group, the serum from cynomolgus monkeys vaccinated with the recombinant vaccine significantly delayed death in mice. All the in vivo experiments validated the non-inferiority of the recombinant vaccine.

In summary, we compared the immunogenicity, the growth and decline of specific antibodies, and the protection of a recombinant tetanus vaccine and the currently available tetanus toxoid vaccine in mice, rats, and cynomolgus monkeys. The recombinant tetanus vaccine showed excellent efficacy as a booster and represents a potential candidate to enhance adolescent and adult immunity as an alternative to the currently available toxoid vaccine.

## 4. Materials and Methods

### 4.1. Vaccines, Toxin, Toxoid, and Protein

The recombinant tetanus vaccine was produced in our laboratory, containing 20 μg/dose/0.5 mL TeNT-Hc and 1 mg/dose/0.5 mL Al (OH) 3 as adjuvant. The commercial tetanus toxoid vaccine (Lot No: 201205002-2), which is based on inactivated tetanus toxin adsorbed to aluminum hydroxide adjuvant, with a volume of 5mL and the vaccine potency above 40IU per 0.5 mL per human dose was purchased from Shanghai Institute of Biological products Co. Ltd, Shanghai, China. The TT protein contained in the commercial vaccines should be more than 20 μg, which corresponds to about 7 µg TeNT-Hc. The tetanus neurotoxin and toxoid were obtained from the National Institute for Food and Drug Control. The TeNT-Hc protein was expressed and purified in our laboratory as described previously [16]. The LD50 in mice and rats was determined by the improved Karber method. The LD50 of tetanus neurotoxin was about 15.8 ng/kg in mice and 22.8 ng/kg in rats.

### 4.2. Animals

Female, specific pathogen-free (SPF) BALB/c mice, weighing 18 g to 20 g each, were purchased from Beijing Vital River Laboratory Animal Technology Co. Ltd, Beijing, China. Male SPF Wistar rats, weighing about 200 g each, were purchased from Beijing Vital River Laboratory Animal Technology Co. Ltd, Beijing, China. Cynomolgus monkeys, half-male and half-female, each weighing 3 kg to 5 kg, were purchased from Nanning Fuzee Wild Animal Breeding Co. Ltd, Nanjing, China. All animals were raised under humanitarian conditions. All animal experiments used in this study were performed according to the protocols approved by the Institutional Animal Care and Use Committee of Beijing Institution of Biotechnology (Identification code for mice: IACUC of AMMS-08-2014-005; Date of approval: 18 November 2014. Identification code for rats: IACUC of AMMS-08-2014-006; Date of approval: 20 November 2014. Identification code for cynomolgus monkeys: ACU14-419; Date of approval: 26 June 2014).

### 4.3. Antibodies and Other Reagents

The goat anti-mouse IgG conjugated with peroxidase and the rabbit anti-rat IgG conjugated with peroxidase were purchased from Abcam (Cambridge, UK). The rabbit anti-monkey IgG conjugated with peroxidase was purchased from Sigma-Aldrich (St. Louis, MO, USA). All other chemicals and reagents were obtained from other commercial sources and were of the highest purity available.

### 4.4. Immunization of Mice and Rats for Long-Term Serological Comparison

All 6 BALB/c mice or Wistar rats were classified in one group. Each mouse or rat was immunized subcutaneously (sc) with a 0.5-mL dose of one of the two vaccines (the recombinant tetanus vaccine and the tetanus toxoid vaccine), respectively. The immunized mice or rats were bled every week after the first immunization for serological testing. The tail tip of each mouse or rat was amputated, and the serum of approximately 0.1 mL was collected into a 0.5-mL Eppendorf tube. A second vaccination was performed once the anti-TeNT-Hc or anti-TT antibody titers decreased. Serum samples were collected from tails after the second immunization until Week 44. The serum samples of rats were collected until Week 42.

### 4.5. Immunization of Cynomolgus Monkeys

Each of the ten cynomolgus monkeys (half male and half female) was classified under a single group. Each monkey received an intramuscular injection of 0.5 mL containing one of the two vaccines every three weeks. Venous blood of the immunized monkeys was collected before each immunization for serological testing. Two and four weeks after the fifth vaccination, the serological tests were also performed.

### 4.6. Immunization of Mice for Comparison of Booster Vaccination Efficacy

Two experiments were carried out to evaluate the efficacy of booster vaccination using the two kinds of vaccines in mice.

#### 4.6.1. Booster at Week 7 Using a Different Vaccine

Twelve SPF BALB/c mice were classified into two groups. Each BALB/c mouse was immunized abdominally and subcutaneously (s.c.) with 0.5 mL of the tetanus toxoid vaccine. Seven weeks after the first immunization, mice in one group underwent a second vaccination with the recombinant tetanus vaccine and the other group received the second vaccination with the tetanus toxoid vaccine. The immunized mice were bled at Weeks 6, 8, 12, 16, 20, 24, 28, 30, 34, 38, 42, and 46 for serological testing.

#### 4.6.2. Booster at Week 28 Using a Different Vaccine

Twelve SPF BALB/c mice were classified into two groups. Each BALB/c mouse was immunized subcutaneously (s.c.) and intra-abdominally with 0.5 mL of the tetanus toxoid vaccine at Weeks 0 and 7. Mice in one group received the third vaccination with the recombinant tetanus vaccine, and the other group received the third vaccination with the tetanus toxoid vaccine at Week 28. The immunized mice were bled at Weeks 4, 8, 12, 16, 20, 24, 28, 30, 34, 38, 42, and 46 for serological testing.

### 4.7. ELISA

Using a formalin-inactivated tetanus toxoid or purified TeNT-Hc as the solid-phase antigen to measure the titer of the antibody induced by the recombinant tetanus vaccine or the tetanus toxoid vaccine, 96-well plates (Costar) were coated with 2 μg/mL and 50 μL/well of antigen in PBS at 4 °C and incubated overnight. They were blocked for 1 h with 3% BSA. The serum samples collected from mice or rats or cynomolgus monkeys were two-fold serially diluted from 1:100 to 1:409,600 in PBS and then added to the plate and incubated for 2 h at 37 °C. The plates were washed with PBST and then incubated with a 1:5000 dilution of peroxidase-labeled goat anti-mouse total IgG antibody or a peroxidase-labeled rabbit anti-rat total IgG antibody or a peroxidase-labeled rabbit anti-monkey total IgG antibody for 1 h at 37 °C. The plates were washed again, and the peroxidase substrate, tetramethylbenzidine (TMB, Millipore Sigma, Beijing, China), was added to each well and incubated at room temperature. The TMB reaction was stopped with 2 M H2SO4, and the absorbance at 450 nm was read with a microplate reader (Bio-Rad, Beijing, China).

### 4.8. Mice and Rats Challenged Long after Vaccination

Mice and rats, which were immunized twice as described in Section 2.4 were challenged with tetanus neurotoxin 44 and 42 weeks after the first vaccination, respectively, by intraperitoneal injection of 2 × 10^3^ 50% lethal doses (LD50s) of toxin in 0.5 mL in borate-buffered saline (0.5 g of borax, 4.5 g of boric acid, and 8.5 g of sodium chloride in 1 L of distilled water). Mice and rats were observed for 10 days, and those alive were scored as survivors.

### 4.9. Potency Comparison of Low-Dose Vaccines in Mice

To further compare the potency of the recombinant tetanus vaccine and the tetanus toxoid vaccine, the two kinds of vaccines were 1000-fold diluted with normal saline. Each BALB/c mouse (6 per group) received an intraperitoneal injection of diluted vaccines in 0.5 mL portions at Weeks 0, and 4. Serum samples were collected from tails four weeks after the first vaccination and two weeks after the second vaccination, respectively. The anti-TeNT-Hc IgG titers and anti-TT IgG titers were analyzed using ELISA as described in Section 4.7. All mice were challenged with 100 LD50s of tetanus neurotoxin at Week 6 and observed for 10 days, and those alive were scored as survivors. One group of mice was immunized with PBS and the adjuvant as a negative control.

### 4.10. Serological Neutralizing Activity of the Vaccinated Cynomolgus Monkeys

To compare the neutralizing activities of the antibodies induced by the two kinds of vaccines in cynomolgus monkeys, the serum of the cynomolgus monkeys immunized four weeks after the last time as described in Section 2.5 were pooled and tested. Twenty LD50s of tetanus neurotoxin were mixed with 0.5 μL of serum and incubated at 37 °C for 0.5 h. After incubation, the mixtures were injected intraperitoneally (ip) into the BALB/C mice (8 per group). All mice were monitored for 10 days, and those alive were scored as survivors. One group of mice, which was only challenged with 20 LD50s of tetanus neurotoxin, was used as the negative control.

## Figures and Tables

**Table 1:** In vivo protection of the recombinant tetanus vaccine and the tetanus toxoid vaccine in mice and rats.

Animals	Vaccine	Numbers of Animals	Tetanus Toxin Challenge (LD50s)	Survival Rate
mice	recombinant tetanus vaccine	6	2 × 10^3^	6/6
	tetanus toxoid vaccine	6	2 × 10^3^	6/6
	negative control	6	2 × 10^3^	0/6
rats	recombinant tetanus vaccine	6	2 × 10^3^	6/6
	tetanus toxoid vaccine	6	2 × 10^3^	6/6
	negative control	6	2 × 10^3^	0/6

## Abbreviations

The following abbreviations are used in this manuscript:

TeNT	tetanus neurotoxin
TeNT-Hc	Hc domain of tetanus neurotoxin
TT	tetanus toxoid
SPF	specific pathogen-free
TMB	tetramethylbenzidine
DPT	diphtheria pertussis tetanus
